# Management and outcome of patients supported with Impella 5.0 for refractory cardiogenic shock

**DOI:** 10.1186/s13054-015-1073-8

**Published:** 2015-10-09

**Authors:** Philippe Gaudard, Marc Mourad, Jacob Eliet, Norddine Zeroual, Geraldine Culas, Philippe Rouvière, Bernard Albat, Pascal Colson

**Affiliations:** Department of Anesthesiology and Critical Care Medicine, Arnaud de Villeneuve Hospital, CHRU Montpellier, 371 Avenue du Doyen Gaston Giraud, 34295 Montpellier, France; PhyMedExp, University of Montpellier, INSERM U1046, CNRS UMR9214, 371 avenue du Doyen G. Giraud, 34295 Montpellier, France; Department of Cardiac Surgery, Arnaud de Villeneuve Hospital, CHRU Montpellier, 371 Avenue du Doyen Gaston Giraud, 34295 Montpellier, France

## Abstract

**Introduction:**

Cardiogenic shock refractory to standard therapy with inotropes and/or intra-aortic balloon pump is accompanied with an unacceptable high mortality. Percutaneous left ventricular assist devices may provide a survival benefit for these very sick patients. In this study, we describe our experience with the Impella 5.0 device used in the setting of refractory cardiogenic shock.

**Methods:**

In this observational, retrospective, single-center study we included all the consecutive patients supported with Impella 5.0, between May 2008 and December 2013, for refractory cardiogenic shock. Patients’ baseline and procedural characteristics, hemodynamics and outcome to the first 48 h of support, to ICU discharge and day-28 visit were collected.

**Results:**

A total of 40 patients were included in the study. Median age was 57 years and 87.5 % were male. Cardiogenic shock resulted from acute myocardial infarction in 17 patients (43 %), dilated cardiomyopathy in 12 (30 %) and postcardiotomy cardiac failure in 7 (18 %). In 15 patients Impella 5.0 was added to an ECMO to unload the left ventricle. The median SOFA score for the entire cohort prior to circulatory support was 12 [10–14] and the duration of Impella support was 7 [5–10] days. We observed a significant decrease of the inotrope score (10 [1–17] vs. 1 [0–9]; *p* = 0.04) and the lactate values (3.8 [1.7–5.9] mmol/L vs. 2.5 [1.5–3.4] mmol/L; *p* = 0.01) after 6 h of support with Impella 5.0. Furthermore, at Impella removal the patients’ left ventricular ejection fraction improved significantly (*p* < 0.001) when compared to baseline. Cardiac recovery, bridge to left ventricular assist device or heart transplantation was possible in 28 patients (70 %). Twenty-six patients (65 %) survived at day 28. A multivariate analysis showed a higher risk of mortality for patients with acute myocardial infarction (hazard ratio = 4.1 (1.2–14.2); *p* = 0.02).

**Conclusions:**

Impella 5.0 allowed fast weaning of inotropes and might facilitate myocardial recovery. Despite high severity scores at admission, day-28 mortality rate was better than predicated.

## Introduction

Cardiogenic shock (CS) remains a clinical challenge, with high mortality rates. Mechanical circulatory support (MCS) is increasingly used, whereas inotropes are associated with adverse events, inadequate circulatory supply or high myocardial oxygen consumption [1]. Data demonstrating the benefit of MCS in CS and helping guide the choice between the different available devices are needed [2]. A recent trial has demonstrated that intra-aortic balloon pump (IABP) did not improve outcomes of patients with CS complicating myocardial infarction (CSMI) [3, 4]. Peripheral venoarterial extracorporeal membrane oxygenation (PVA-ECMO) presents major advantages including quick and easy implantation, oxygen supply, biventricular assistance and good systemic blood flow; moreover, it is a cost-effective therapy. The PVA-ECMO can be used in various etiologies of CS [5] and seems to be the appropriate support for persistent cardiac arrest [6], biventricular failure and emergency MCS in remote institutions [7]. However, PVA-ECMO can be limited by insufficient left ventricular (LV) unloading resulting in thrombus formation or pulmonary edema, especially in case of profound LV failure [8, 9].

The Impella 5.0 device (Abiomed Europe GmbH, Aachen, Germany) provides a continuous blood flow up to 5 L/min and is designed to support patients in CS due to isolated LV failure for 10 days. Recent data have suggested that percutaneous left ventricular assist devices (LVADs) may provide a survival benefit for patients with refractory CS after myocardial infarction [10] or cardiotomy [11–13]. Experimental data have demonstrated that Impella 5.0 improved recovery after myocardial infarction [14]. Optimal LV unloading, which procures optimal myocardial oxygen supply/demand ratio, is better achieved with Impella, with or without associated PVA-ECMO [14, 15]. The use of Impella 2.5 devices (but not of Impella 5.0) for LV unloading in case of severe LV distension for patients on PVA-ECMO was previously described in case reports [16–19] and seems to be a promising therapeutic option.

The purpose of the current report was to describe our results with the Impella 5.0 device, used either as full LV support or complementary to PVA-ECMO in refractory CS, on cardiac recovery or successful switch to long-term LVAD or heart transplantation, ICU outcome, and day-28 mortality.

## Materials and methods

### Patients

We retrospectively reviewed the MCS database of our 14-bed intensive care unit (ICU) from May 2008 (first case of Impella 5.0 use) to December 2013 to register all patients implanted with Impella 5.0. During the study period, among 178 MCS cases recorded, the first-line support strategy for refractory CS was Impella 5.0 for 25 patients (14 %). The decision for support with Impella 5.0 first was taken by the physician on duty if the hemodynamic status of the patient with isolated LV failure was compatible with surgical implantation. The other 153 patients had had a PVA-ECMO first, and among them, LV unloading was required with Impella 2.5 (n = 14) or Impella 5.0 (n = 15; 10 % of PVA-ECMO patients). The cases with Impella 2.5 or PVA-ECMO alone are not described in this report.

The study was approved by the Montpellier University Hospital institutional review board, which, because of the retrospective nature of the study, waived the need for informed consent.

### Definitions

Refractory CS was defined by evidence of tissue hypoxia (high blood lactate level, low venous oxygen saturation) concomitant with sustained hypotension and reduced cardiac index below 2.2 L/min/m^2^ despite adequate intravascular volume and optimal dose of inotropes and vasopressors. Isolated LV failure was defined by severely altered LV function (LV ejection fraction below 20 %) and no or mild right ventricular (RV) dysfunction at echocardiographic evaluation. Severe LV overload during PVA-ECMO support was considered in case of severe pulmonary edema, acute LV dilatation and/or visualization of spontaneous contrast in left heart cavities on echocardiography since PVA-ECMO support was set at the minimal level to cover metabolic needs as assessed by central venous oxygen saturation (ScvO_2_) and lactate. The beginning of MCS (t0MCS) matched with the time of first MCS implantation with either Impella 5.0 or PVA-ECMO. Sustained cardiac recovery was defined by a patient without inotropes or MCS or transplant at ICU discharge.

### Mechanical circulatory support systems and implantation

All devices were implanted by an experienced cardiac surgeon. The Impella 5.0 device (Abiomed Europe GmbH, Aachen, Germany) is a 9 F catheter-mounted microaxial intracardiac pump (21 F), surgically inserted in the femoral artery (arteriotomy) or the right axillary artery (through a vascular graft) in the operating room or in the cardiac catheterization laboratory. It is positioned across the aortic valve into the left ventricle using fluoroscopy and/or transesophageal echocardiography and provides up to 5 L/min continuous blood flow by transvalvular active support with direct LV unloading. In Europe, this device is approved for short-term use, up to 10 days. PVA-ECMO was implanted in the operating room when transfer of the patient was possible, if not in the ICU or catheterization laboratory, and consisted of polyvinyl chloride tubing with a membrane oxygenator (PH.I.S.I.O and EOS; Sorin Group, Clamart, France), a centrifugal pump (Stockert; Sorin Group), and percutaneous or surgically inserted arterial and venous femoral cannulae (Fem-Flex and Fem-Track, Edwards Lifesciences, Guyancourt, France) with an additional 7 F cannula inserted distally into the femoral artery to prevent lower limb ischemia.

### Patient management during Impella 5.0 support

Echocardiographic evaluation was systematically performed during initial settings, after each modification and before explantation of the device. Unfractionated heparin in hypertonic glucose solution (as recommended by the manufacturer) was administered through the Impella device to maintain an activated partial thromboplastin time between 1.5 and 2 times the normal value. For Impella-alone patients, the pump speed was adjusted to obtain sufficient blood flow for the oxygen demand with the lowest possible dose of inotropes. The RV function was assessed daily with echocardiography and enhanced if needed using vasopressors, inotropes, pacing, inhaled nitric oxide (iNO) to decrease RV afterload, and depletion. In case of persistent circulatory shock, especially because of RV failure, PVA-ECMO was added to the Impella device for global circulatory support. When Impella was secondarily combined with PVA-ECMO, the pump speed of Impella was adjusted in order both to unload LV and to prevent upper body hypoxia at the initial phase. After resolution of pulmonary edema, Impella flow was favored over PVA-ECMO flow and PVA-ECMO weaning was performed first, if possible.

The weaning process was left to the discretion of the physician guided by daily echocardiographic evaluation. Impella 5.0 weaning was performed in a stepwise fashion by decreasing the pump speed with echocardiography monitoring. Once the performance level of the device was reduced to the lowest level to avoid forward flow for 2 h, with stable cardiac index, mean arterial pressure (MAP) and ScvO_2_, the device was removed in the operating room. When MCS weaning was not possible, bridging to LVAD or heart transplantation was considered, provided there was no severe multiorgan failure.

### Data collection

Demographic data and MCS characteristics were collected: age, sex, etiology of CS, Impella 5.0 implantation site, cardiac arrest before MCS, transfer on MCS from other centers, time from onset of CS to t0MCS, time from t0MCS to Impella 5.0 implantation for PVA-ECMO patients. The following clinical variables were collected at ICU admission and t0MCS: Simplified Acute Physiology Score II (SAPS II) [20], Sepsis-related Organ Failure Assessment (SOFA) score [21], MAP, invasive mechanical ventilation (MV) support, IABP support, inotropic support (dobutamine and/or epinephrine infusion) and blood lactate.

Before initiation, at ICU return (hour 0) and during the first 48 h of Impella 5.0 support, the following data were recorded: the vasoactive-inotropic score as defined as dose of dobutamine (μg/Kg/min) + [dose of epinephrine (μg/Kg/min) + dose of norepinephrine (μg/Kg/min)] x 100, and the inotrope score defined as dose of dobutamine (μg/Kg/min) + [dose of epinephrine (μg/Kg/min)] x 100 [22, 23], MV support, partial pressure of oxygen in arterial blood (PaO_2_) to inspired oxygen fraction (FiO_2_) ratio, MAP, blood lactate, N-terminal pro-brain natriuretic peptide (NT-proBNP) levels, Impella 5.0 and PVA-ECMO flows. Cardiothoracic ratios were calculated from digital chest radiographs (ratio between the maximum cardiac transverse diameter and the maximum thoracic diameter measured between the inner margins of the ribs).

Duration of Impella and PVA-ECMO support, occurrence of severe RV failure (needing additional PVA-ECMO) when Impella was used alone, PVA-ECMO weaning on Impella 5.0 support, MV weaning during the Impella course, incidence of transfusion and numbers of red blood cells (RBC) units transfused during MCS were also recorded.

### Complications and outcome variables

Complications associated with Impella 5.0 were reported: major device malfunction (device failure), minor device malfunction (flow, position or pressure monitoring failure), device malposition (intra-aortic or intra-LV moving, successful bedside repositioning), incidence of blood transfusion during surgery, bleeding requiring reoperation, arterial ischemia at implantation site, stroke, hemolysis defined by increase of free bilirubin and lactate dehydrogenase (LDH) with or without anemia unexplained by bleeding (free hemoglobin dosage was not available at this time in our institution), ventricular arrhythmia, and device-related infection.

The main outcome variables included death before Impella removal, cardiac outcome in Impella survivors (sustained cardiac recovery; bridge to LVAD; bridge to heart transplantation), day-28 mortality, ICU mortality, and month-6 mortality. The other ICU outcome variables were the MV duration, the need for renal replacement therapy (RRT), and the length of ICU stay.

### Statistical analysis

All data are presented as absolute values and percentages (%) for categorical variables or median and interquartile range (IQR) for continuous data. Paired Wilcoxon test was used to describe evolution of clinical and biological variables during first 48 h of Impella 5.0 support. Kaplan-Meier curves, with 28 days follow-up, were plotted to show the survival trend of patients according to the etiology of CS. Log-rank test was used to compare the survival difference between acute myocardial infarction and other etiologies. We performed a Cox proportional hazards regression model to identify variables that significantly influenced day-28 mortality, as measured by the hazard ratio with the 95 % confidence interval (CI). Variables related to mortality in the univariate analysis with *p* value < 0.2 were further analyzed in a stepwise multivariable Cox model. Statistical significance was defined as *p* value < 0.05. Analyses were performed using XLSTAT 2013 software (Addinsoft, New York, NY, USA).

## Results

### Study population

Baseline characteristics of the 40 patients (35 males, 87.5 %) who were supported by Impella 5.0 device for refractory CS are displayed in Table 1. Etiologies of CS were in order of frequencies: acute ST-elevation myocardial infarction (43 %), end-stage dilated cardiomyopathy (30 %), postcardiotomy (18 %) and others like myocarditis or contusion (10 %). Time from onset of CS to initiation of MCS (t0MCS) was 24 h (IQR 12–47). Twenty-five patients (62.5 % of the Impella 5.0 cohort) were treated by first intention with Impella 5.0 alone including three who had an additional PVA-ECMO. For one of them, PVA-ECMO was indicated for hypoxic cardiac arrest occurring during myocardial recovery and caused by acute respiratory distress syndrome in a patient with posttraumatic myocardial contusion. For the two other patients, MCS was upgraded for RV failure during Impella support (dilated cardiomyopathy and one myocarditis). Impella 5.0 was initiated in 15 patients (37.5 %) on PVA-ECMO support to unload the left ventricle and/or a switch strategy to a specific LV support. The median time between the beginning of extracorporeal support and Impella implantation was 20 h (IQR 7–45). Patients were younger but more severe at the time of MCS initiation in the subgroup of patients with Impella combined with PVA-ECMO versus patients supported by Impella first as assessed by SOFA score (respectively 14 [IQR 12.5–16] vs. 10 [IQR 8–12], *p* < 0.001) and blood lactate value (respectively 6 [IQR 5.1–12.6] vs. 3.4 [1.7–4.4], *p* = 0.008).

**Table 1:** Baseline characteristics of patients supported with the Impella 5.0 device

Patients characteristics	Total (n = 40)
Gender (female/male)	5/35
Age, years	57 [48–63]
Surgical access femoral/axillary	30/10
Etiology of cardiogenic shock	
Acute ST-elevation myocardial infarction	17 (43)
Dilated cardiomyopathy	12 (30)
Postcardiotomy	7 (18)
Others	4 (10)
Context of MCS	
Cardiac arrest before MCS	9 (23)
Transfer from others centers with MCS	4 (10)
Severity at ICU admission	
SOFA score	11 [9–14]
SAPS II score	58 [46–76]
Severity and treatment at t0MCS	
Mean arterial pressure, mmHg	60 [50–68]
SOFA score	12 [10–14]
Blood lactate, mmol/l	3.8 [1.7–5.9]
IAPB support	11 (28)
Inotrope support	32 (80)
Mechanical ventilation	29 (73)
MCS description	
Impella 5.0 alone	25 (62.5 %)
Impella 5.0 added to ongoing ECMO	15 (37.5 %)
Duration of Impella support, days	7 [5–10]
Duration of ECMO, days	5 [3–8]
Total duration of MCS, days	7 [5–10]

Data are median [interquartile range] or absolute values (%)

*MCS* mechanical circulatory support, *ICU* intensive care unit, *SOFA* Sepsis-related Organ Failure Assessment, *SAPS II* Simplified Acute Physiology Score II, *t0MCS* time of first initiation of MCS, *IABP* intra-aortic balloon pump, *ECMO* extracorporeal membrane oxygenation

### Mechanical circulatory support settings and clinical course

**Fig. 1:**
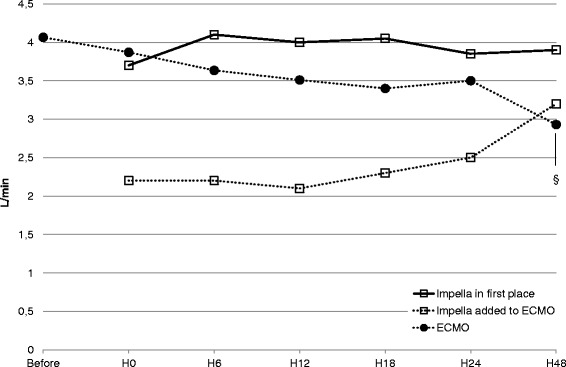
Variations of mechanical circulatory support flow (median values) before and during the first 48 h of Impella support. Impella flow is represented by *empty squares* and ECMO flow by *full circles*. ^§^ *p* < 0.05 for comparison with H0 flow, paired Wilcoxon test. *ECMO* extracorporeal membrane oxygenation

**Fig. 2:**
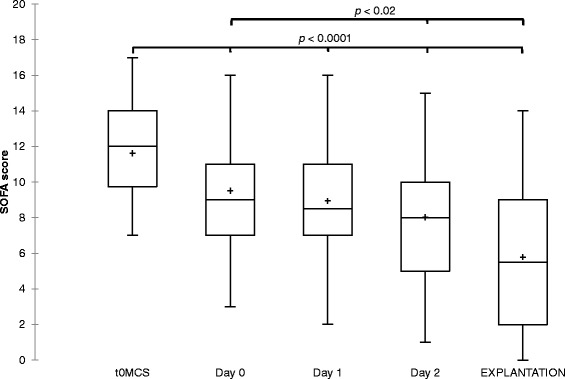
Box plots of serial monitoring of the SOFA score, before mechanical circulatory support (t0MCS), during the first 48 h of Impella support and at the day of Impella removal. The *line* in the box indicates the median value of the data and the *cross* indicates the mean value. Paired Wilcoxon test for comparison with values at t0MCS and at day 0. *SOFA* Sepsis-related Organ Failure Assessment

The flows of Impella and PVA-ECMO during the first 48 h were represented separately for Impella alone and Impella combined in Fig. 1. The ECMO flow was significantly reduced between hour 0 and hour 48 (3.9 [3.3–4.7] L/min versus 2.9 [2.4–3.6] L/min; *p* < 0.01) as the Impella flow tended to increase only when combined with PVA-ECMO (2.2 [1.8–3.1] L/min vs. 3.2 [2–3.9] L/min; *p* = 0.29). The clinical course, pump flows of MCS and hemodynamic parameters are summarized in Table 2. The use of inotropes was withdrawn early as assessed by the decrease of inotrope score from 10 [1–17] before implantation to 1 [0–9] at hour 6 (*p* = 0.04) while the blood lactate level decreased from 3.4 [1.7–5.1] mmol/L at hour 0 to 2.5 [1.5–3.4] mmol/L at hour 6 (*p* < 0.01). Significant improvement in the multiorgan dysfunction syndrome were observed during the first 2 days of Impella support as assessed by serial monitoring of the SOFA score (Table 2 and Fig. 2). Cardiac parameters of global overload like cardiothoracic ratio and NT-proBNP level were also decreased at hour 24 when compared to before Impella implantation (Table 2). Seven patients out of 25 on Impella 5.0 alone (28 %) were weaned from MV with a successful extubation during Impella support but none out of the 15 patients already on PVA-ECMO at the implantation time. Ten patients out of 15 (67 %) could be weaned from PVA-ECMO on Impella with a median time between Impella implantation and PVA-ECMO removal of 4 days (IQR 3–5).

**Table 2:** Clinical course and cardiac parameters before and during the first 48 h of support with Impella 5.0

	Before implantation	ICU return	6 hours	24 hours	48 hours
Alive, number (%)	40 (100 %)	40 (100 %)	39 (98 %)	38 (95 %)	37 (93 %)
MAP, mmHg	65 [58–74]	76 [66–86]^*a*^	76 [71–80]^a^	79 [74–88]^*a*^	80 [75–86]^*a*^
Impella flow, L/min	-	3.5 [2.6–3.9]	3.8 [2.4–4.3]	3.7 [2.5–4.2]	3.9 [3.3–4.2]^*b*^
ECMO flow, L/min	4.1 [2.5–4.3]	3.9 [3.3–4.7]	3.6 [3.5–4.2]	3.6 [3.1–4.1]^*b*^	3.2 [2.5–3.9]^*b*^
PaO2/FiO2 ratio	204 [160–311]	188 [121–280]	203 [132–324]	245 [187–327]	244 [172–335]
ScvO2^*^, %	62 [56–68]	74 [68–77]	70 [65–77]	75 [70–80]	70 [69–73]
Blood lactate (mmol/L)	3.8 [1.7–5.9]^**^	3.4 [1.7–5.1]	2.5 [1.5–3.4]^*a,b*^	1.6 [1.2–2.8]^*a,b*^	1.5 [1.0–2.1]^*a,b*^
SOFA score	12 [9.8–14.0]^**^	9.0 [7.0–11.0]^*a*^	-	8.5 [7.0–11.0]^*a,b*^	8.0 [5.0–10.0]^*a,b*^
Vasoactive-inotropic score	51 [13–112]	47 [17–96]	43 [18–82]	24 [8–56]^*a,b*^	9 [0–32]^*a,b*^
Inotrope score	10 [1–17]	5 [0–15]	1 [0–9]^*a,b*^	0 [0–4]^*a,b*^	0 [0–0.4]^*a,b*^
Cardiothoracic ratio	0.58 [0.52–0.66]	-	-	0.55 [0.50–0.60]^*a*^	0.54 [0.49–0.59]^*a*^
NT-proBNP (ng/L)	3736 [1436–8024]	-	-	1638 [799–5689]^*a*^	1780 [745–3931]^*a*^

Data are median [interquartile range]

*MAP* mean arterial pressure, *ECMO* extracorporeal membrane oxygenation, *PaO2* partial pressure of oxygen in arterial blood, *FiO2* inspired fraction of oxygen, *ScvO2* central venous oxygen saturation, *SOFA* Sepsis-related Organ Failure Assessment, *NT-proBNP* N-terminal pro-brain natriuretic peptide

^*^Fifteen (37.5 %) patients were monitored with ScvO2

^**^Values recorded before mechanical circulatory support

^*a, b*^Indicates *p* value < 0.05 compared to values before Impella implantation and at ICU return after Impella implantation respectively, paired Wilcoxon test

### Complications

Some complications were reported during Impella support (Table 3). In four patients, a major device malfunction resulted in stopping the circulatory support: one case at the ICU returned after PVA-ECMO removal with cardiac arrest despite no apparent anomaly on the Impella monitor (massive acute aortic regurgitation suspected, fatal issue), one case due to electric damage on the pump after transfer to the operating room for PVA-ECMO removal associated with hemorrhagic shock due to accidental ablation of the arterial cannula (fatal issue), and an unexplained technical problem for two of them (one after 5 h, the other one after 9 days of normal running) but nonfatal because of sufficient cardiac recovery. The other clinically relevant adverse events were: device-related infection (18 %), device displacement (20 %) and evidence or suspicion of hemolysis (10 %). The incidence of RBC transfusion was high: 30 patients (75 %) needed transfusion of at least one unit of RBC during the MCS period.

**Table 3:** Incidences of complications or adverse events during Impella 5.0 support

Type of complication	Values
Major device malfunction, n (%)	4 (10)
Minor device malfunction, n (%)	3 (8)
Device displacement (intra-aortic or intraventricular moving), n (%)	8 (20)
Including successful bedside repositioning, n	6
Bleeding requiring transfusion during surgical implantation, n (%)	7 (18)
Bleeding requiring surgery after implantation, n (%)	0 (0)
RBC transfusion on MCS, units [IQR]	4 [1.5–8]
RBC transfusion by day on MCS, units [IQR]	0.4 [0.1–1.2]
Upper or lower limb ischemia on implantation site, n (%)	1 (3)
Thromboembolic events, n (%)	1 (3)
Major hemolysis, n (%)	1 (3 %)
Suspected or minor hemolysis, n (%)	3 (8 %)
Ventricular arrhythmia, n (%)	3 (8)
Device-related infection, n (%)	7 (18)
Surgical site infection, n (%)	4 (10)
Infected thrombus on the head of the pump, n (%)	3 (8)
Bloodstream infection during MCS, n (%)	5 (13)

Major hemolysis: anemia without bleeding associated with an increase of free bilirubin and lactate dehydrogenase (LDH). Suspected or minor hemolysis: low increase of free bilirubin and LDH without unexplained anemia

*RBC* red blood cells, *MCS* mechanical circulatory support, *IQR* interquartile range

### Clinical outcome

**Fig. 3:**
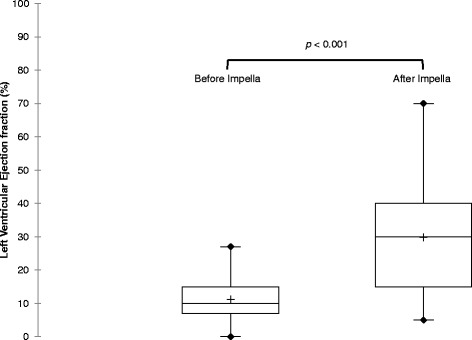
Box plots of left ventricular ejection fraction measured before Impella 5.0 implantation and after Impella removal during ICU stay. The *line* in the box indicates the median value of the data and the *cross* indicates the mean value. Paired Wilcoxon test for comparison. *ICU* intensive care unit

Left ventricular ejection fraction was significantly improved from 10 % (IQR 7–10) before implantation to 30 % (IQR 15–40) after Impella removal, *p* < 0.001 (Fig. 3). Cardiac outcome, main ICU management and mortality are reported in Table 4. Successful Impella weaning was achieved in 18 patients (45 % of total population, 64 % of survivors during Impella support) but two of them were implanted with an LVAD before ICU discharge because of remaining LV failure. In case of impossible weaning, direct transition to LVAD and heart transplantation was performed for seven and three patients respectively. None of the nine patients who underwent LVAD after Impella showed a RV failure after implantation.

**Table 4:** Cardiac, clinical and global outcomes

Event	Total (n = 40)
Death during Impella 5.0 support, n (%)	12 (30)
Impella 5.0 weaning, n (%)	18 (45)
Sustained cardiac recovery^a^ at ICU discharge, n (%)	16 (40)
Bridge to LVAD, n (%)	9 (23)
Bridge to heart transplantation, n (%)	3 (8)
Total MV duration, days [IQR]	15 [7–26]
Need for RRT in ICU, n (%)	17 (43)
ICU length of stay, days [IQR]	20 [8–32]
Mortality at day 28, n (%)	14 (35)
Post-AMI, n (%)	9 (53 %)
Dilated cardiomyopathy, n (%)	3 (25 %)
Postcardiotomy, n (%)	1 (14 %)
Others, n (%)	1 (25 %)
Mortality at ICU discharge, n (%)	17 (43)
Mortality at month 6, n (%)	20 (50)

*ICU* intensive care unit, *LVAD* left ventricular assist device, *MV* mechanical ventilation, *IQR* interquartile range, *RRT* renal replacement therapy, *AMI* acute myocardial infarction

^a^Defined by ICU discharge without inotropes or mechanical circulatory support or transplant

**Fig. 4:**
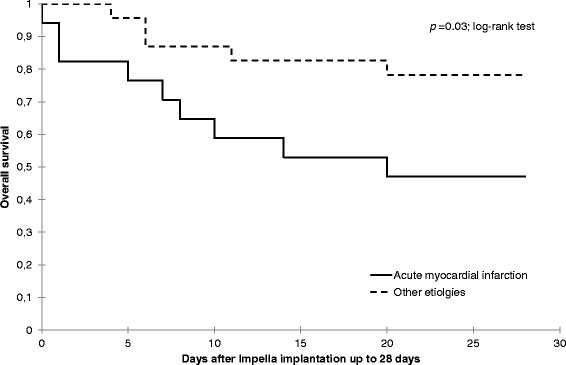
Kaplan Meier analysis of day-28 survival for patients with cardiogenic shock complicating myocardial infarction versus other etiologies (dilated cardiomyopathy, postcardiotomy cardiogenic shock, miscellaneous)

The mortality rate at day 28 was 35 % with a significant lower survival rate for CS complicating myocardial infarction when compared to other etiologies (47 % vs. 88 %; Khi^2^ test: *p* = 0.04), also shown on the Kaplan-Meier analysis (Fig. 4). No differences in outcome and in mortality were found between patients with Impella first and those with combination with PVA-ECMO.

### Overall predictors of day-28 mortality

From the univariate analysis, SAPS II score at ICU admission, the rate of RBC transfusion by day on MCS and the acute myocardial infarction etiology were associated with day-28 mortality (Table 5). Stepwise multivariate model revealed that myocardial infarction etiology, vasoactive-inotropic score before Impella implantation and SAPS II score were independent risk factors for the day-28 mortality. Patients who had CS following acute myocardial infarction had a higher risk of mortality (hazard ratio = 4.1, 95 % CI (1.2–14.2); *p* = 0.02).

**Table 5:** Predictors of day-28 mortality in univariate and stepwise multivariate Cox regression analysis

	Univariable		Stepwise multivariable	
	hazard ratio (95 % CI)	*p*	hazard ratio (95 % CI)	*p*
SOFA score at t0MCS	1.16 (0.95–1.4)	.14	-	-
Cardiac arrest before MCS	2.35 (0.79–7.03)	.13	-	-
RBC transfusion by day on MCS	1.91 (1.17–3.12)	.01	-	-
RRT during ICU stay	2.13 (0.74–6.14)	.16	-	-
SAPS II score at ICU admission	1.04 (1.01–1.07)	.01	1.04 (1.01–1.07)	0.008
Vasoactive-inotropic score before Impella	1.00 (0.998–1.01)	.18	1.01 (1.00–1.02)	.035
Post-AMI cardiogenic shock	3.06 (1.02–9.16)	.05	4.14 (1.20–14.25)	.024
Cardiogenic shock from dilated cardiomyopathy	0.57 (0.16–2.06)	.40	Not included	
Postcardiotomy cardiogenic shock	0.32 (0.04–2.44)	.27	Not included	
Cardiogenic shock from other etiologies	0.61 (0.08–4.63)	.63	Not included	

All patient variables related to mortality in univariate analysis, defined by *p* < 0.2 and cardiogenic shock etiologies subgroups are reported. Variables with *p* ≥ 0.2 were not included in the model. The first four variables entered into the model were not independently associated with mortality in the stepwise multivariable model

*SOFA* Sepsis-Related Organ Failure Assessment, *t0MCS* time of first initiation of MCS, *MCS* mechanical circulatory support, *RBC* red blood cells, *RRT* renal replacement therapy, *ICU* intensive care unit, *SAPS II* Simplified Acute Physiology Score II, *AMI* acute myocardial infarction

## Discussion

We report herein our experience with the Impella 5.0 device for patients in refractory CS of various etiologies, either as single LV support or as LV unloading on previous PVA-ECMO. To date, only a few studies have described the use of the Impella 5.0 device in CS, almost exclusively after cardiotomy [12, 13]. To our knowledge, it is the largest series of LV unloading with an Impella 5.0 device for patients on PVA-ECMO. Our single-center cohort of 40 Impella 5.0 implantations is original through its management of circulatory support going to a more selective strategy with an efficacy end point, which includes cardiac recovery or bridge to other therapy.

As a main result of this study, a low day-28 mortality rate of 35 % was observed in a population of refractory CS with significant and early improvement of LV ejection fraction in survivors after Impella removal when compared to before implantation. Second, Impella 5.0 allowed a sustained recovery of cardiac function in 40 % or a bridge to LVAD or urgent heart transplantation in 30 % of implanted patients. Finally, the Impella 5.0 device provided an efficient hemodynamic supply in refractory CS even if used alone and transition from PVA-ECMO to Impella 5.0 was possible in most patients with combined support.

In case of refractory CS, comparison between the different devices is difficult because of the lack of comparative trials. Published data [10, 24] suggest that survival was better when using Impella 5.0 compared to 2.5 devices for CS complicating myocardial infarction (CSMI). Recent studies [11–13] have reported a very good survival in case of postcardiotomy CS (PCCS) compared to historical series using Impella LD [25] or ECMO [26]. Less hemorrhagic complications and a better selection of patients more likely to benefit from this technique may explain these results. However, to date, the superiority of Impella 5.0 devices has not been proven and PVA-ECMO represents a more cost-effective device [27]. Whereas Impella 5.0 could be useful in selected patients presenting with CS due to isolated LV failure, the relative position of this device compared to other MCS systems needs to be clarified. PVA-ECMO is the unique option when RV function is impaired or in case of persistent cardiac arrest [6]. Because it allows full cardiac and respiratory supplies, PVA-ECMO also seems an appropriate choice for the most severe patients. However, an interesting potential advantage of Impella is the reduction of inotropes use. These treatments are associated with a worse prognosis of acute cardiac failure [28, 29]. In our series, the quick decrease of inotrope score was observed within 6 h after starting Impella and was withdrawn within 24 h for most patients. Another potential benefit of Impella is to unload the LV. Effective LV unloading without inotropes during Impella assistance was assessed by the decrease in NT-proBNP levels and cardiothoracic ratio within 24 h. We strongly believe that this LV unloading, which may prevent LV remodeling, and the restrictive use of inotropes are relevant advantages when compared to other techniques in this clinical setting. Improvement of myocardial recovery by Impella 5.0 device after acute myocardial infarction is already supported by experimental data [14, 30], especially versus PVA-ECMO [15]. Functional LV recovery was previously observed with Impella 2.5 in less severe LV failure [31] but clinical data with Impella 5.0 are missing. This is the first report that describes a major improvement of LV ejection fraction from very profound LV failure during Impella 5.0 support. Moreover, LV distension is a threatening complication of PVA-ECMO. It is the result of major LV failure and increased LV afterload because of the retrograde aortic reinjection of PVA-ECMO. The incidence and risk factors of this complication are not well described, in fact. Management of LV distention during PVA-ECMO is not standardized and various techniques have been described [8]. Because of complications with conventional techniques (atrial septotomy, ECMO centralization), interest in minimally invasive techniques such as IABP [32] and Impella devices is growing [16–19]. Using Impella 5.0 offers a larger range of flow than Impella 2.5, one reason to choose the largest device to unload the LV when a switch strategy was conceivable. Previous studies have already suggested a survival benefit of the use of Impella 5.0 compared to 2.5 devices for CSMI in small series [10, 24]. In our registry, LV distension occurred in the early phase of PVA-ECMO support as assessed by the period of 20 h between PVA-ECMO and Impella implantations. Moreover, successful transition from PVA-ECMO to Impella 5.0 support, achieved in 67 % cases including eight out of ten patients (80 %) who survived to short-term MCS, suggests that ECMO duration can be shortened while patients can still benefit from LV assistance with Impella. Median duration of PVA-ECMO was only 5 days, shorter than that described by Combes et al. (7 days for ICU survivors) in a comparable series of CS of various etiologies [5].

To date, few studies have described adverse events with Impella 5.0 device [11, 24]. However, as any effective MCS like IABP and ECMO, Impella 5.0 is an invasive technique requiring a surgical vascular approach of major arteries and is thus associated with several risks. Very few ischemic complications were observed, that is in agreement with the results of a meta-analysis on percutaneous LVAD [33]. A recurrent complication is bleeding and the increased need for RBC transfusion with a potential impact on mortality [34, 35]. As described by Loforte et al. [36], incidence of transfusion is high during PVA-ECMO, up to 100 %. In the present series, 75 % of patients were transfused. The high incidence of transfusion may be related to hemolysis and bleeding at the surgical site. Hemolysis occurred or was suspected in 10 % of our patients but was easily decreased when pump speed was reduced. Of note, a high incidence of device-related infection was observed, due to the bad clinical status of these patients, as usually observed in severe ICU patients. Not surprisingly, contamination of the device is then a possible complication, which was resolved after Impella removal in most cases. One major concern in this series is the occurrence of major device malfunctions leading to a fatal issue for two patients but some associated factors responsible for death were found: aortic regurgitation and hemorrhagic shock.

If the main goal during MCS for refractory CS remains the myocardium recovery, a bridge to LVAD can be a surrogate goal in younger patients when cardiac recovery is not possible. In this study, Impella 5.0 support allowed a bridge to LVAD for nine patients. Interestingly, none of these patients had presented a severe RV failure (defined by the need of RV mechanical support) after LVAD implantation. These results are surprisingly good when considering that about 20 % of patients requires RV support after LVAD implantation [37, 38]. In fact, if RV evaluation is crucial before LVAD implantation [39], based on hemodynamic and echocardiographic variables, it is a challenging problem in patients in CS because of the low cardiac output due to the LV failure or the unloaded RV by PVA-ECMO can conceal the ability of the RV to cope with the flow coming from a left ventricle assistance [40–42]. In this respect, Impella 5.0 support, as a first therapy or after PVA-ECMO, reproduces the real conditions of LVAD and allows an optimal evaluation of the RV function.

The day-28 mortality, the rate of ICU death and month-6 mortality were 35 %, 43 % and 50 % respectively, whereas severity scores at ICU admission predicated a mortality rate more than 60 %. A previous study in 2008 found an ICU mortality rate at 58 % for patients supported by ECMO for refractory CS [5]. Another retrospective study regarding less severe CS without initial indication for MCS except IABP reported a 30 % day-28 mortality rate [43]. Interestingly, our results confirm those previously published with Impella 5.0: two series in PCCS [12, 13], one in CSMI [10], one for CSMI and PCCS [11]. A higher risk of day-28 mortality was found in a multivariate analysis for patients with CSMI (Table 5). In a previous study with Impella 5.0, a lower survival rate was also reported for CS in the settings of myocardial infarction or dilated cardiomyopathy compared to PCCS [11]. One hypothesis to explain this difference should be the sudden and profound onset of CS following an extended myocardial infarction. The time from onset of CS to Impella 5.0 implantation is probably too long in these very poor circulatory conditions. A specific heart team with ICU physicians may provide an early recognition of patients rapidly progressing to refractory CS during percutaneous coronary interventions. High doses of vasopressors and inotropes before Impella implantation, as described by the vasoactive-inotropic score, were independently associated with higher mortality. This finding, supported by previous publications [11, 44], speaks in favor of MCS over escalating doses of medical treatment. To summarize about factors associated to mortality, SAPS II score at ICU admission and vasoactive-inotropic score before Impella are independently associated with mortality. We can speculate that Impella failure occurs more likely when shock is too much advanced. Although post-acute myocardial infarction cardiogenic shock is an independent factor associated with day-28 mortality, we can hypothesize that these patients have potential high benefits from support by Impella (high flow supply and LV unloading, with improved coronary blood flow and decreased myocardial workload). We strongly believe that an early decision for Impella implantation in this post-acute myocardial infarction population, before a high increase of vasopressors and inotropes treatment, may contribute to improved survival. This parameter should be taken into account in future comparative trials.

Our study has several limitations. First of all, as this is not a controlled trial with a comparison group versus Impella, all data presented can only help to generate hypotheses but cannot prove a survival benefit of Impella 5.0 support in these patients. Although the retrospective, single-center nature, and the sample size of this study are important limitations, to our knowledge, it is one of the largest series about Impella 5.0 utilization. The use of Impella 5.0 to unload the left ventricle associated with a transition strategy for patients on PVA-ECMO is a new description of ICU management during CS. Nevertheless, selection of patients who can benefit from this strategy among all patients on PVA-ECMO was not controlled and should be an important bias. Furthermore, although the indication of Impella 5.0 support in the first place was based on clinical and echocardiographic evaluation, some patients with isolated LV failure were probably supported with PVA-ECMO for many reasons like Impella monitor or operating room availabilities. Unfortunately, details on echocardiographic data like cardiac output and LV diameters are missing because echocardiographic examinations were not always securely recorded and only LV ejection fraction was systematically reported in medical charts. Another possible limitation is mixing various etiologies of cardiogenic shock, but the major characteristic of the hemodynamic failure originates from a primary cardiac failure; therefore, pump dysfunction is the common feature that requires similar treatment despite the various mechanisms.

## Conclusions

This retrospective, observational study reports that Impella 5.0 support indicated for refractory cardiogenic shock was associated with a low day-28 mortality rate and led to myocardial recovery, transplantation or long-term support in 70 % of the implanted patients. Besides, some promising characteristics of Impella 5.0 support like fast weaning of inotropes, preservation of transpulmonary blood flow and optimal RV evaluation in the prospective of LVAD are suggested. Impella 5.0 seems a good mechanical assistance in the early phase of cardiogenic shock, when circulatory failure is not too critical. In acute ischemic conditions, it may protect against left ventricle remodeling, but further studies are needed to confirm long-term myocardial recovery and left ventricle function improvement.

## Key messages

- Day-28 mortality rate was 35 % after circulatory support with Impella 5.0 for refractory CS
- Left ventricular ejection fraction improved from 10 to 30 % during Impella support
- Sustained cardiac recovery or successful transition to LVAD or heart transplantation were obtained for 70 % of implanted patients

## Abbreviations

CI: confidence interval
CS: cardiogenic shock
CSMI: cardiogenic shock complicating myocardial infarction
FiO_2_: inspired oxygen fraction
IABP: intra-aortic balloon pump
ICU: intensive care unit
iNO: inhaled nitric oxide
IQR: interquartile range
LDH: lactate dehydrogenase
LV: left ventricular
LVAD: left ventricular assist device
MAP: mean arterial pressure
MCS: mechanical circulatory support
MV: mechanical ventilation
NT-proBNP: N-terminal pro-brain natriuretic peptide
PaO_2_: partial pressure of oxygen in arterial blood
PCCS: postcardiotomy cardiogenic shock
PVA-ECMO: peripheral venoarterial extracorporeal membrane oxygenation
RBC: red blood cells
RRT: renal replacement therapy
RV: right ventricular
SAPS II: Simplified Acute Physiology Score II
ScvO_2_: central venous oxygen saturation
SOFA: Sepsis-related Organ Failure Assessment
t0MCS: time of first initiation of MCS
